# Oral Hygiene Practices and Teeth Cleaning Techniques Among Medical Students

**DOI:** 10.7759/cureus.1487

**Published:** 2017-07-18

**Authors:** Sajida Naseem, Syeda H Fatima, Haider Ghazanfar, Sana Haq, Najeeb A Khan, Moeez Mehmood, Ali Ghazanfar

**Affiliations:** 1 Department of Community and Family Medicine, Shifa International Hospital, Islamabad, Pakistan; 2 Department of Health Profession Education, Shifa College of Medicine-Stmu Islamabad ,pakistan; 3 Internal Medicine, Newark Beth Israel Medical Center; 4 Family Medicine, Alam Hospital, Pakistan; 5 Federal Medical and Dental College, Islamabad, Pakistan

**Keywords:** oral hygiene, practices, primary health, oral health, medical student, gender, healthy lifestyle habits

## Abstract

Objectives

Oral health is essential for general health and quality of life. It is a state of being free from mouth and facial pain, oral and throat cancer, oral infections and sores, periodontal disease, tooth decay, tooth loss, and other diseases and disorders that limit an individual’s capacity to bite, chew, smile, and speak; it affects psychosocial well-being too. The objective of our study was to assess teeth cleaning techniques and oral hygiene practices among medical students.

Methods

The data of the study were collected in two stages. The first stage involved the administration of a self-constructed questionnaire among medical students. In the second step, the students were asked to demonstrate their teeth cleaning techniques on a model. A standard teeth cleaning checklist was used to evaluate the students. The students were then given the checklist and a video on teeth cleaning techniques was shown to them. The data obtained was analyzed on IBM's statistical package for the social sciences (SPSS) version 21.

Results

Out of a total of 444 students, 256 (57.7 percent) were males while 188 (42.3 percent) were females. About 254 (57.2 percent) participants were preclinical medical students while 190 (42.8 percent) were clinical year medical students. A majority of medical students used medium consistency toothbrushes (177; 39.9 percent) and soft consistency toothbrushes (137; 30.9 percent). Most medical students (248; 55.9 percent) brushed two times a day while 163 (36.7 percent) brushed only one time. About 212 (47.7 percent) of the medical students used mouthwash along with a toothbrush while only 36 (8.1 percent) used floss along with a toothbrush. About 157 participants (35.4 percent) changed their toothbrush once in two months while 132 (26.7 percent) changed their toothbrush once in three months. The mean duration that participants brushed their teeth was 134.99 ± 69.01 seconds.

Conclusion

Medical students were found to have a faulty teeth cleaning technique. There is a dire need to spread awareness about correct teeth cleaning techniques because poor oral hygiene can have a detrimental effect on the overall health and quality of life of an individual.

## Introduction

Oral health is an essential component of a person’s health. According to World Health Organization (WHO), about 60 percent to 90 percent of children and nearly every adult in the world have dental cavities [1]. According to American Dental Association (ADA), “oral health is a functional, structural, aesthetic, physiologic, and psychosocial state of well-being and is essential to an individual’s general health and quality of life” [2]. 

Poor oral health is related to significant morbidity and mortality [3]. According to a study done in the USA, it was concluded that the frequency of emergency department visits because of preventable dental conditions has increased by 16 percent since 2006 [4]. Poor oral hygiene has a significant impact on general health and is associated with various systemic diseases [5]. The systemic diseases also have an impact on oral diseases. According to a review article, poor glycemic control is associated with an increased risk of severe periodontitis [6]. A study done on Indian students concluded that teenagers were unable to correctly assess their dental hygiene. The authors attributed this to a lack of oral health self-assessment skills, which resulted in poor oral hygiene [7].

Considering the fact that medical students are more knowledgeable and aware of health problems, we hypothesized that medical students would be able to maintain appropriate dental hygiene. It is important to assess their oral health knowledge, attitude, and practice because they are future professionals who will be responsible for managing and preventing oral health diseases. The objectives of our study were to assess the teeth-cleaning techniques and oral hygiene practices among medical students.

## Materials and methods

We performed an analytical cross-sectional study among medical students studying at Shifa College of Medicine, a private medical education institution in Pakistan, from June 2016 to December 2016. A self-constructed questionnaire consisting of 28 items was used to assess the teeth cleaning technique and oral hygiene practices among medical students. All medical students studying in Shifa College of Medicine during this time period were eligible to participate in the study. The students were divided into two groups. The pre-clinical group consisted of students studying in the first, second, and third years while the clinical group consisted of students studying in the fourth and final years.

Informed consent was taken from all participants and the identity of the respondents was kept anonymous. The data of the study were collected in two stages. The first stage involved the administration of a self-constructed questionnaire among medical students. In the second step, students were asked to demonstrate teeth cleaning techniques on a model. A standard teeth cleaning checklist was used to evaluate the students. The students were then given the checklist and a video on teeth cleaning techniques was shown to them.

The data obtained was analyzed on IBM's statistical package for the social sciences (SPSS) version 21. An independent t-test was done to see if there was any statistically significant association (p<0.05) of gender and year of medical education with the score on the teeth cleaning demonstration and the mean number of soft drinks consumed. A Spearman’s correlation was run to determine the association of the duration of teeth brushing of the participants with the score on the teeth cleaning demonstration. A p-value less than 0.05 was considered significant. The Mann-Whitney test was applied to determine the association of prior knowledge of teeth cleaning guidelines with the score on the teeth cleaning demonstration. A p-value less than 0.05 was considered statistically significant. The Kruskal-Wallis H test was used to determine the association of the number of times teeth are brushed per day with the score on the teeth cleaning demonstration. Ethical approval for the study was obtained from Shifa International Hospital's review board, ethical committee IRB number: 430-279-2015.

## Results

Out of a total of 444 students, 256 (57.7 percent) were males while 188 (42.3 percent) were females. A total of 254 participants (57.2 percent) were preclinical medical students while 190 (42.8 percent) were clinical year medical students. The majority of medical students (177; 39.9 percent) used either medium consistency toothbrushes and soft consistency toothbrushes (137; 30.9 percent). About 18 medical students (4.1 percent) did not know which type of toothbrush they used. Most medical students (248; 55.9 percent) brushed two times a day while 163 (36.7 percent) brushed only one time a day. About 212 (47.7 percent) of the medical students used mouthwash along with a toothbrush while only 36 (8.1 percent) used floss along with a toothbrush. When asked which ingredient the participants preferred to use in toothpaste, 262 (59.1 percent), 99 (22.3 percent), and 83 (18.7 percent) participants preferred fluoride, salt, and mint, respectively. Most participants (241; 54.3 percent) had come across teeth cleaning guidelines. About 157 participants (35.4 percent) changed their toothbrush every two months while 132 (26.7 percent) changed their toothbrush once every three months. The mean duration of teeth brushing of participants was 134.99 ± 69.01 seconds.

The majority of the medical students (339; 76.4 percent) went to a dentist only when needed. About 114 (25.7 percent) stated that their teeth were sensitive to hot/cold while 42 (9.5 percent) stated that their teeth were discolored. Around 78 medical students (17.6 percent) had discolored teeth and about 69 (15.5 percent) stated they had tooth caries. Around 96 participants (21.6 percent) smoked cigarettes while 75 (16.9 percent) smoked “*shisha*” regularly. Only six participants (1.4 percent) ate "*paan*" (a combination of betel leaf with areca nut and tobacco) regularly. Around 85 participants (19.1 percent) brushed after consuming sweets. The mean number of soft drinks consumed by the participants in a weeks' time was 5.51 ± 4.48.

Only 21 (4.7 percent) held the toothbrush at 45 degrees while cleaning their teeth. About 138 (31.1 percent) performed circular movements of the toothbrush while 128 (28.8 percent) performed circular movements inside outside and only 47 (10.6 percent) slid the brush down after each circular movement. Only nine participants (2.0 percent) got all four steps right. Most of the participants, 286 (64.4 percent), did not get a single step right.

An independent t-test was done to see if there was the statistically significant difference (p<0.05) between the gender, the score on the teeth cleaning demonstration, and the mean number of soft drinks consumed in a week’s time. Female participants were found to have a greater score on the teeth cleaning demonstration as compared to male participants (p=0.001). On the other hand, male participants were found to consume more soft drinks per week as compared to female participants (p<0.001). This has been presented in Table 1.

**Table 1:** Association of Gender with Oral Hygiene Practices and Teeth Cleaning Techniques

Factors	Gender	Mean+S.D	F	p-value
Score on the teeth cleaning demonstration	Male	0.598+0.997	28.442	0.001
	Female	0.963+1.225		
Mean number of soft drinks consumed in a weeks' time	Male	6.820+4.852	14.672	<0.001
	Female	3.712+3.156		

An independent t-test was done to see if there was the statistically significant association (p<0.05) between the year of medical education, the score on the teeth cleaning demonstration, and the mean number of soft drinks consumed in a weeks' time. Medical students in the clinical year were found to have a greater score on the teeth cleaning demonstration than preclinical year students (P=0.024). This has been shown in Table 2.

**Table 2:** Association of Year of Medical Education with Oral Hygiene Practices and Teeth Cleaning Techniques

Factors	Year of Medical Education	Mean+S.D	F	p-value
Score on the teeth cleaning demonstration	Preclinical	0.650+1.040	10.997	0.024
	Clinical	0.890+1.192		
Mean number of soft drinks consumed in a weeks' time	Preclinical	5.661+4.167	0.157	0.401
	Clinical	5.300+4.881		

A Spearman’s correlation was run to determine the association of the duration of teeth brushing of the participant with the score on the teeth cleaning demonstration. No statistically significant correlation was found (rs = -0.093, p = 0.050). The Mann-Whitney test was applied to determine the association of prior knowledge about teeth cleaning guidelines with the score on the teeth cleaning demonstration. Participants with prior knowledge of teeth cleaning guidelines scored statistically significantly higher than the participant group who had no prior knowledge of teeth cleaning guidelines (U=20309, p<0.001). The Kruskal-Wallis H test was used to determine the association of the number of times teeth are brushed per day with the score on the teeth cleaning demonstration. The Kruskal-Wallis H test showed that there was a statistically significant difference in the score on the teeth demonstration between the number of times teeth were brushed per day, χ2(2) = 14.492, p = 0.001, with people brushing two times a day having the highest score on the teeth demonstration and people brushing only one time a day having the lowest score. 

## Discussion

Numerous studies have been carried out to investigate gender-based differences in oral health. The prevalence of caries has been higher in women than in men, and the finding has been documented across various cultures and time periods [8]. Many factors can be attributed to this disparity, including lifestyle variations and cultural norms [9]. However, according to a study done on Greek dental students, who were exposed to a literate, urban environment and had better knowledge of health care, females were more likely than their male counterparts to maintain oral hygiene. This could be explained by the fact that women are more conscious of their appearance and body image than men and, consequently, have a proactive approach to promote and maintain oral health [10]. According to a study done in eastern India, it was concluded that female participants had a higher score in terms of knowledge and practices of oral health as compared to their male counterparts [11]. According to a study done in Jordan, it was concluded that female students brushed their teeth more frequently than male students (p<0.01) did. The study also concluded that male students were more likely to smoke than female students ( 31 percent vs. 4 percent) and, as a result, had poorer oral hygiene (p<0.01) [12]. A study done in northeast Thailand reported that female students had positive health habits as compared to male students [13]. In our study, the difference in the scores of female and male students for the teeth cleaning demonstration was statistically significant. Though both female and male students had similar educational backgrounds, women scored higher than men and this could be attributed to the importance they place on body image. Female students also consumed a fewer number of soft drinks than male students did. The more positive health behavior of female students is a result of several factors, including the importance of appearance and awareness about health maintenance. 

Our study also found a statistically significant difference in the score on the teeth cleaning demonstration as the years of medical education increased. Students in their senior clinical years had a higher score than students attending preclinical classes. Greater health awareness and increased exposure to the clinical side of medicine could account for the higher score in students in their clinical years. However, though senior medical students did consume less soft drinks than their junior counterparts, the difference in the number of beverages consumed was not statistically significant. Because students in their clinical clerkships educate and counsel their patients on various health promotion behaviors, including nutrition counseling, it is expected that as the academic year advances, students become more self-aware about their own deleterious attitudes to health [14]. A study done in India showed that final-year medical students had a significantly higher score for oral health knowledge, attitude, and behavior than first-year medical students (p<0.01) [15]. A study in Nigeria concluded that older students had better oral health, attitude, knowledge, and practice as compared to younger students [16]. The results of our study are important in devising programs to improve oral health among medical students. This can have a ripple effect, as students who practice good oral hygiene personally would be able to counsel their patients more effectively. 

## Conclusions

Medical students were found to have a faulty teeth cleaning technique. There is a dire need to spread awareness about correct teeth cleaning techniques because poor oral hygiene can have a detrimental effect on the overall health and quality of life of an individual.
